# The Enzymatic Paradox of Yeast Arginyl-tRNA Synthetase: Exclusive Arginine Transfer Controlled by a Flexible Mechanism of tRNA Recognition

**DOI:** 10.1371/journal.pone.0148460

**Published:** 2016-02-04

**Authors:** Ariel McShane, Eveline Hok, Jensen Tomberlin, Gilbert Eriani, Renaud Geslain

**Affiliations:** 1 Laboratory of tRNA biology, Department of Biology, College of Charleston, Charleston, South Carolina, United States of America; 2 Architecture et Réactivité de l'ARN, Université de Strasbourg, CNRS, Institut de Biologie Moléculaire et Cellulaire, 15 rue René Descartes, 67084, Strasbourg CEDEX, France; Max-Planck-Institute for Terrestrial Microbiology, GERMANY

## Abstract

Identity determinants are essential for the accurate recognition of transfer RNAs by aminoacyl-tRNA synthetases. To date, arginine determinants in the yeast *Saccharomyces cerevisiae* have been identified exclusively *in vitro* and only on a limited number of tRNA Arginine isoacceptors. In the current study, we favor a full cellular approach and expand the investigation of arginine determinants to all four tRNA Arg isoacceptors. More precisely, this work scrutinizes the relevance of the tRNA nucleotides at position 20, 35 and 36 in the yeast arginylation reaction. We built 21 mutants by site-directed mutagenesis and tested their functionality in YAL5, a previously engineered yeast knockout deficient for the expression of tRNA Arg CCG. Arginylation levels were also monitored using Northern blot. Our data collected *in vivo* correlate with previous observations. C35 is the prominent arginine determinant followed by G36 or U36 (G/U36). In addition, although there is no major arginine determinant in the D loop, the recognition of tRNA Arg ICG relies to some extent on the nucleotide at position 20. This work refines the existing model for tRNA Arg recognition. Our observations indicate that yeast Arginyl-tRNA synthetase (yArgRS) relies on distinct mechanisms to aminoacylate the four isoacceptors. Finally, according to our refined model, yArgRS is able to accommodate tRNA Arg scaffolds presenting N34, C/G35 and G/A/U36 anticodons while maintaining specificity. We discuss the mechanistic and potential physiological implications of these findings.

## Introduction

Aminoacyl-tRNA synthetases (AARSs) participate in the translation of the genetic code by covalently linking specific amino acids to the 3’ of appropriate transfer RNAs (tRNAs) [1]. AARSs recognize specific features on their cognate tRNAs that are defined as identity elements or determinants [2] [3]. Determinants are absolutely essential for the recognition by cognate AARS and are absent in non-cognate tRNAs. Identity elements are typically embedded in the primary sequence using ribonucleotides (i.e., ACGU), but materialize occasionally through transcriptional modifications [4–6]. The contributions of idiosyncratic local 2D structures and post-transcriptional modification are challenging to assess in contrast to identity nucleotides, which can be conveniently and individually probed by site directed mutagenesis [7]. Consequently, identity nucleotides have been extensively scrutinized and mapped on numerous tRNAs in various species [7]. This vast body of work indicates that, in the majority of tRNAs, identity nucleotides are clustered in the anticodon and the acceptor stem.

Arginine, leucine and serine are notable exceptions in the genetic code as these amino acids are encoded by six different codons. As a consequence, tRNA Arg, Leu and Ser display complementary anticodons that are highly variable in primary sequence. The set of identity elements for these particular tRNAs had to extend beyond the anticodon and the acceptor stem in order to maintain a strict specificity [8]. In tRNA Leu and tRNA Ser, the unique structural feature of the large variable arm serves as a complementary identity element [9–11].

Arginine determinants were first investigated and identified in *E*. *coli* through cellular suppression assays and *in vitro* aminoacylation experiments involving synthetic tRNA variants [2, 3, 12]. In bacterial tRNA, major arginine determinants are limited to three nucleotides in two distinct regions: the C35 and G/U36 in the anticodon loop and the highly conserved A20 in the D loop. The discriminatory bases G or C73 contribute only modestly to the recognition by *E*. *coli* ArgRS and are considered as auxiliary determinants [13].

**Fig 1:**
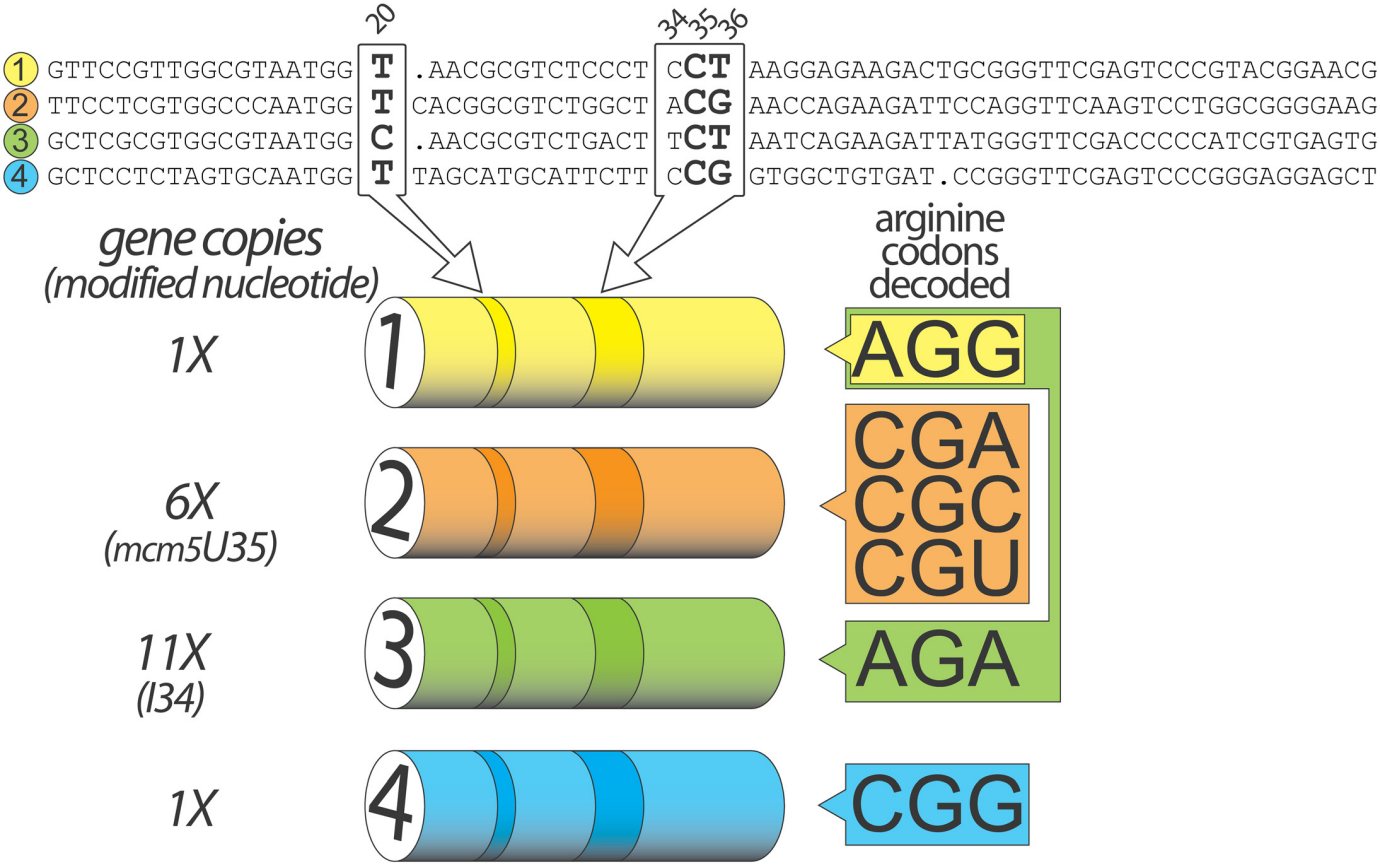
The nuclear genome of S. cerevisiae encodes four different tRNA Arg genes. In yeast, nineteen genes encode four different tRNA Arg: tR1, 2, 3 and 4. The figure indicates for each isoacceptor gene, the primary sequence, the number of copies and the arginine codon(s) decoded by the corresponding transcript. Two areas of the tRNA Arg are particularly relevant for this study and are highlighted on the cartoon: nucleotide 20 in the D loop and nucleotides 35 and 36 in the anticodon loop. Both color codes and highlighted positions are consistent throughout the different figures. _mcm5_U34 in tR3 stands for 5-methoxycarbonylmethyluridine.

The nuclear genome of *Saccharomyces cerevisiae* encodes four different tRNA Arg isoacceptors displaying CCU, ICG, _mcm5_UCU, and CCG anticodons. In the current study, these tRNA are abbreviated as tR1, 2, 3 and 4 respectively (Fig 1). A previous report demonstrated that C35 and G/U36 are essential for the aminoacylation of tR3 transcripts by yeast Arginyl-tRNA synthetase (yArgRS) confirming the model for anticodon recognition established in *E*. *coli* [14]. However, the bacterial model failed to explain how natural yeast tRNA Arg displaying exclusively C20 or U20 in place of the conserved A20 could be recognized by yArgRS. The yeast arginylation system was *de facto* assimilated as evolutionary divergent and considered as the exception that proves the rule. The two-pronged recognition mechanism involving the D-loop and the anticodon in bacteria has been thereon reduced to a one-pronged mechanism in yeast.

In the current study, we expanded the investigation of arginine determinants to all four tRNA Arg isoacceptors in *S*. *cerevisiae*. We favored a cellular approach that integrates fundamental cellular parameters such as the presence of post-transcriptional modifications and the notion of competition for the access to aminoacyl-tRNA synthetases. Our approach, based on the expression of tRNA Arg variants *in vivo*, capitalizes on previously engineered genetic tools. To gain further insight into the allegedly one-pronged recognition mechanism, we focused our efforts on the two areas that were previously identified. The ultimate goals were to expose *in vivo* the full extent of, (i) the function of C20 and U20 and (ii) the role of the anticodon in the arginylation reaction.

First, we probed the function of the nucleotides at position 20 across the four tRNA Arg isoacceptors. We built 9 mutants by site directed mutagenesis and tested their functionality in YAL5, a previously engineered yeast knockout deficient for the expression of tR4. Second, we scrutinized the role played by the nucleotides in the anticodon using tR4 as a model. We built 6 single and 6 double mutants of tR4, expressed the corresponding constructs in YAL5 and monitored their arginylation levels by Northern blot.

Our data collected *in vivo* correlates with previous observations. In tR4, C35 is the prominent arginine determinant followed by G36. In addition, the recognition of tR2 relies to some extent on the nucleotide at position 20 although there is no major arginine determinant in the D loop. This work also refines the existing model for tRNA Arg recognition. Our observations indicate that yArgRS relies on different mechanisms to aminoacylate tR1-4, revealing its complex isoacceptor-dependent nature. Finally, our cellular approach demonstrates for the first time the remarkable flexibility of yArgRS. According to our refined model, yArgRS is able to accommodate tR4 scaffolds presenting N34, C/G35 and G/A/U36 anticodons while maintaining specificity.

## Methods

### Vectors and yeast strains

The *Saccharomyces cerevisiae* strain YBAM2 2n (*ura3-52 lys2-801*^*am*^ *trp1-Δ63 his3-Δ200 leu2-Δ1 ade2-Δ450 ade3-Δ1483*) was previously used for the disruption of the tR4 gene [15]. The resulting YAL5 strain [YBAM2, *tr4*::*HIS3*, pAL5 (tR4^+^, Ura^+^, Ade3^+^)] is rescued by the plasmid pAL5 (Ura^+^, Ade3^+^) encoding the native tR4 gene. YAL5 was transformed with centromeric pRS314 (Trp^+^) or pRS315 (Leu^+^) plasmids encoding various tRNA Arg constructs and grown on selective media according to standard procedures.

### tRNA constructs

A 1.05 kb DNA fragment encoding the tR1 gene and its surrounding sequences was amplified by PCR from genomic DNA and cloned into pRS314. tR2 and tR3 were cloned into pRS314 after the amplification of 0.35 and 0.4 kb fragments respectively. Mutations targeting the D-loop and the anticodon loop were introduced in the different constructs by site directed mutagenesis. A 0.65 kb PCR fragment encoding the tR4 gene has been previously cloned in pRS314 and pRS315 [15].

### Northern blots analysis

Yeast cells expressing mutated tR4 genes were cleared of the pAL5 plasmid (tR4^+^, Ura^+^, Ade3^+^) after transformation with pRS315-tR3 CCG and grown on minimal medium supplemented with adenine (limiting concentration 2 mg/ml), uracil (20 mg/ml) and lysine (20 mg/ml). Yeast cells from white sectors were grown in 100 ml of complete YPD medium to A_700nm_ = 2 and harvested by centrifugation. All subsequent steps were performed on ice. The cell pellet was resuspended in 5 ml of extraction buffer (0.3 M sodium acetate, pH 4.5, 10 mM EDTA) and subjected to two acid-phenol extractions (pH 4.5). Thirty μg of extracted total RNAs were separated on a 1 mm thick 6.5% polyacrylamide gel (19:1, acrylamide:bisacrylamide) containing 8 M urea in 0.1 M sodium acetate buffer pH 5. Electrophoresis was performed at 18 W (400 V) at 4°C for 24 h. Separated RNAs were transferred from the gel onto a nylon membrane (Hybond-XL) and cross-linked at 80°C using a gel dryer. Pre-hybridization was performed for 4 h at 60°C in 40 ml of 1× Denhardt’s solution, 5× SSPE, 0.5% SDS (w/v). Hybridization was performed for 12 h at 60°C in 15 ml of the same solution, in the presence of 5’ ^32^P-labeled DNA probes (20-mers) specific to the 3’ end of tRNA Arg (CCG) and a tRNA Leu (UAA) [15]. Washing was performed in 2× SSPE and 0.5% SDS for 20 min at 50°C. Radioactive signal was quantified by phosphorimaging.

### *In vitro* transcription

tRNAs were *in vitro* transcribed using T7 RNA polymerase from a template generated by primer extension of overlapping DNA oligonucleotides and purified on 10% denaturing polyacrylamide gel. Transcripts were extracted by soaking gel slices overnight at 4°C in 50 mM KOAc and 200 mM KCl, pH 7, precipitated, and resuspended in H_2_O. *In vitro* transcription of tRNA starting with a U, such as tR2, required the use of a leader sequence in order to improve the otherwise poor transcription yield. Different strategies exist for the removal of the transcription-enhancing sequence. The one used in this study consisted of inserting a ribozyme between the leader and the tRNA sequences [16]. Following transcription and proper folding, the autocatalytic RNA released the tRNA that was subsequently purified by gel electrophoresis and elution. Both wild-type tR2 and UC20 mutant were prepared accordingly.

### *In vitro* aminoacylation

Arginylation assays were performed at 37°C in 50 mM HEPES (pH 7.5), 30 mM KCl, 25 μM [^14^C]L -arginine (300 Ci/mol), 10 mM ATP, 10 mM MgCl2, 0.1 mg/ml bovine serum albumin, 5 μM tRNA transcript and 30 nM ArgRS, as described previously.

Threonylation and tryptophanylation assays were performed at 37°C in presence of 25 μM [^14^C]L—threonine and tryptophan respectively and yeast total proteins as a source of ThrRS and TrpRS. Michaelis (Km) and catalytic (kcat) constants were measured following standard procedures [17].

### Mobility shift assays

Dephosphorylated tR2 transcripts (25 pmol) were incubated for 30 min at 37°C with 5 U of T4 polynucleotide kinase (USB Affymetrix) and 25 pmol of 6000 Ci/mmol [γ-^32^P]ATP in 10 μl of dedicated T4 PNK buffer. Labeled tRNA were gel purified as described above.

5’-labeled tR2 transcripts (>10^5^ cpm) were incubated with variable concentrations of purified yArgRS (0.45 to 11.00 μM) for 15 min at room temperature. Complexes were formed in 10 μl of binding buffer containing 25 mM Tris-HCl, pH 7.5, 75 mM KCl, 10 mM MgCl_2_, 25% glycerol and separated at room temperature for 120 min at 120V on native 6% (37.5:1, acrylamide:bisacrylamide) gels (200 x 200 x 1.5 mm^3^). Radioactive signal was quantified by phosphorimaging. Dissociation constants (Kd) were measured following standard procedures [17].

## Results and Discussion

### Chimeric tRNAs as a way to test mutants of tR1, 2, and 3 in YAL5

tRNA arginine CCG (tR4) is encoded by a single nuclear gene. It is absolutely essential for the decoding of CGG codons, which represent 3.9% of the arginine codons used by *S*. *cerevisiae* [15] (Fig 1).

YAL5 is a *S*. *cerevisiae* strain that was originally engineered in our laboratories for the screening of inactive mutants of tR4 [15]. It was successfully used for the *in vivo* identification of key nucleotides involved in the expression, stability and aminoacylation of tR4. YAL5 key genotype alterations comprise: (i) a disrupted and consequently inactive nuclear tR4 gene, (ii) a plasmid (pAL5) encoding both an active copy of tR4 as well as a convenient and unambiguous phenotypic marker and finally (iii) a compatible genetic background enabling the selection of transformants and the expression of the marker.

**Fig 2:**
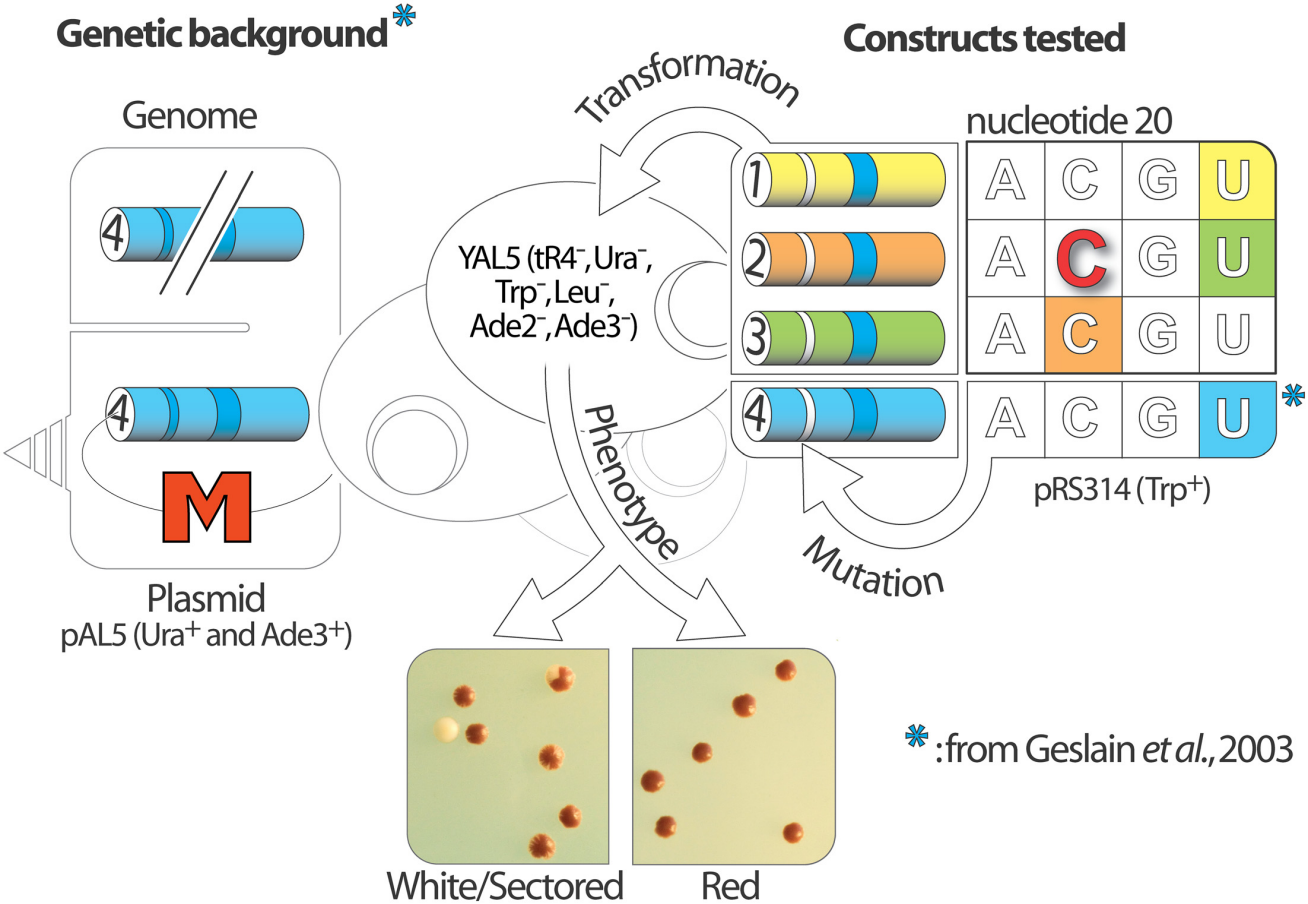
Systematic assessment of nucleotide 20: description of the *in vivo* approach and the phenotypes of the mutants. The left panel summarizes the principle of the genetic tools developed previously and the two observable phenotypes on solid media. The right panel displays, for chimeric tR1, 2 and 3, all the combinations of nucleotide at position 20 that were tested in YAL5. White and red nucleotides indicate functional and inactive mutants respectively. The background color highlights the wild type nucleotide in each tRNA construct.

Specifically, plasmids encoding mutants of tR4 genes are introduced in YAL5 by chemical transformation and plated on selective media. The survival of cells expressing inactive mutants is contingent on the presence of pAL5 and its associated phenotypic marker. The corresponding cells form homogenous red colonies on solid selective media. Alternatively, pAL5 becomes redundant in cells expressing functional mutants and is progressively lost as the cells divide leading to the formation of characteristic white sectors within red colonies [15] (Fig 2).

In a previous report, we demonstrated that U20 in tR4 could be substituted for A, C, or G without any drastic functional impact [15]. Specifically, all three mutants displayed a red/white phenotype in YAL5. For the current study, we conceived and relied on a molecular artifice in order to analyze mutants of tR1-3, through the same colony-sectoring assay. First, the three sequences encoding the corresponding tRNAs and their surrounding sequences were amplified by PCR from genomic DNA and cloned separately in pRS314 plasmids (refer to materiel and methods for details). Second, tR1-3 were turned into CGG decoders; in other words, we harmonized their anticodons to mimic the decoding capacity of tR4. Finally, we validated our strategy by confirming the red/white phenotype and therefore the full activity of the three chimeras in YAL5 (Fig 2).

### Degeneration of nucleotide 20: loss of functionality for only one mutant out of twelve

The three YAL5-compatible chimeras provided suitable scaffolds to study the relevance of position 20 as arginine determinant in tR1-3. Nine mutants were generated via site directed mutagenesis by substituting the original U20 in tR1 and tR2 and C20 in tR3 chimeras for the three other nucleotides. The nine corresponding plasmid-constructs were independently introduced in YAL5 (Fig 2).

The tR2 chimera harboring the UC20 mutation was the only species displaying a solid-red phenotype in YAL5, demonstrating the deleterious effect of this particular nucleotide replacement. Interestingly, the exact same substitution in tR1 and tR4 yielded fully functional tRNA mutants. Moreover, C20 is naturally present in wild-type tR3. These observations challenge a previous universal model for tRNA Arg recognition based on the crystal structure of the tR2/yArgRS complex [18]. It was proposed that the mere flipping of the side chains of residues N106 and Q111 on the protein was sufficient in order to accommodate both U and C20 on the tRNA. According to this model, a UC20 substitution in tR2 should have been fully functional.

Strictly speaking, YAL5 selects tRNA mutants based on their ability to decode CGG codons. In practice, this genetic tool probes indiscriminately every single aspect of the tRNAs in regards to the translation pathway. Ultimate CGG decoding depends intimately on concomitant tRNA expression, aminoacylation and interaction with the ribosome. The D-loop is typically a hot spot for conserved or semi-conserved nucleotides in eukaryotic tRNAs. The A-box, which spans from nucleotide 8 to 19, is directly involved in the recruitment of transcription factors and therefore strongly influences tRNA expression [19]. In addition, nucleotides at position 14, 15, 18 and 19 are consistently involved in long-range tertiary interactions stabilizing the canonical tRNA structure and promoting uniform tRNA binding by the A site of the ribosome [20]. Mutations targeting position 20 are, by definition, innocuous for both the expression and the overall structure of the tRNA variants. Therefore, the functionality of such tRNA mutants assessed through YAL5 boils down to their ability to be charged by yArgRS.

**Fig 3:**
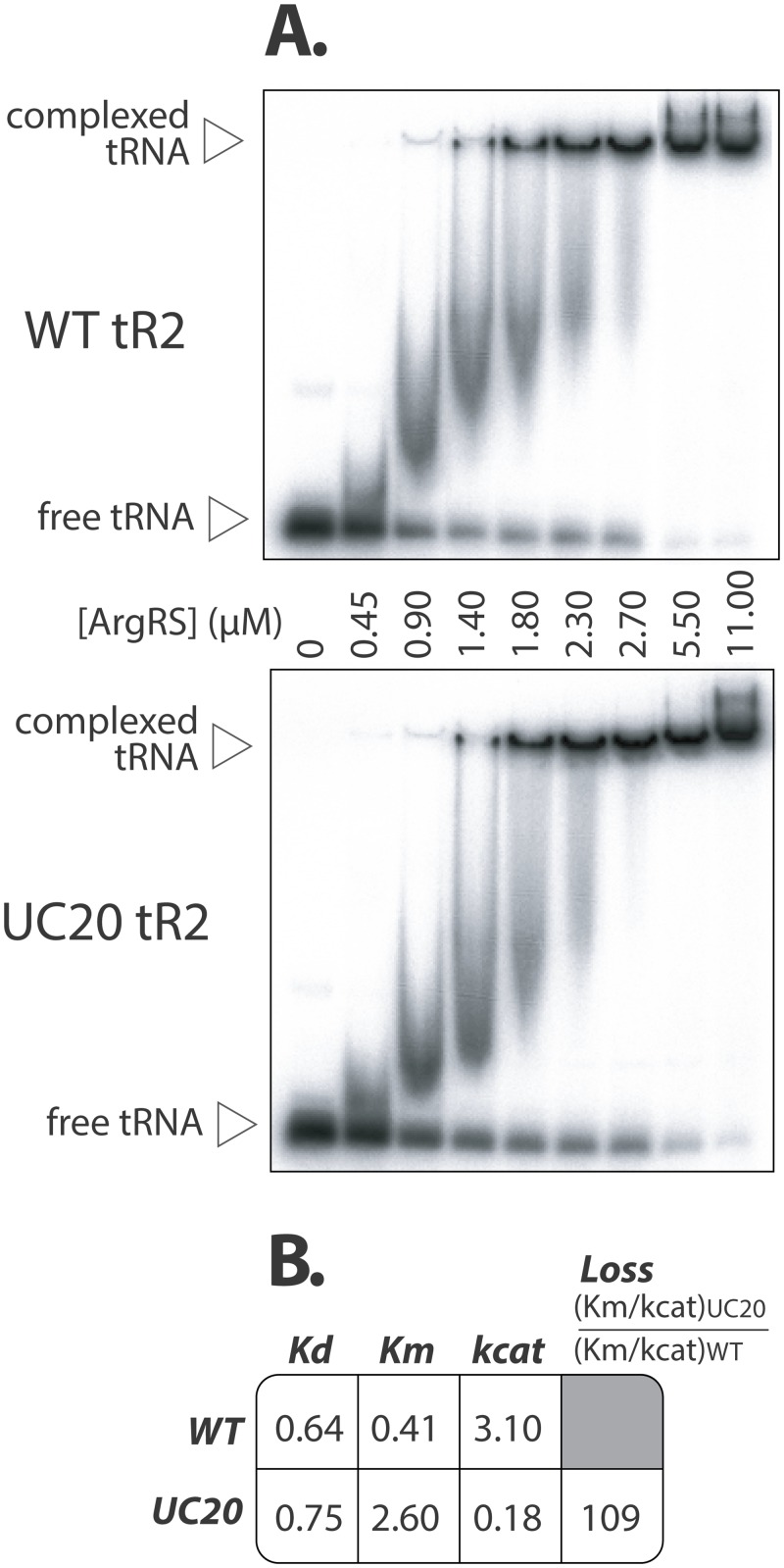
Impact of the UC20 substitution on the binding and the aminoacylation of tR2 by yArgRS. (A) Yeast wild-type tR2 and UC20 mutant were individually transcribed *in vitro*, radiolabeled, incubated with 0 to 11 μM of yArgRS and separated on a 6% non-denaturing polyacrylamide gel. The signals corresponding to free and complexed tRNAs were quantified by phosphorimaging and processed to estimate the protein-tRNA dissociation constant. (B) The same protein-tRNA partners were tested through *in vitro* arginylation assays in order to establish both their Michaelis-Menten and rate equilibrium constants.

It could be reasonably argued that the negative impact of UC20 is circumstantial and apparent only in the context of a chimera. To further examine the effect of this substitution, we synthesized *in vitro* a tR2 transcript harboring a natural ACG anticodon in combination with the UC20 mutation. Control and mutated transcripts were then ^32^P-radiolabeled in 5’ with the polynucleotide kinase and analyzed by electrophoretic mobility shift assay (EMSA) in presence of 0 to 11μM of purified recombinant yArgRS (Fig 3). Control and mutant tR2 showed equivalent dissociation constants of 0.64 and 0.75 μM respectively. However, the mutant was found to be substantially less efficient than the control tRNA in arginylation assays with a loss of Kcat/Km superior at 100 fold (Fig 3).

Altogether, these data reveal for the first time the contribution of the position 20 in the yeast arginylation reaction. The importance of nucleotide 20 is context-dependent and is apparent only in tR2, one of the four arginine-acceptors.

### Nucleotides 35 and 36: apparent flexibility for supposedly strict identity elements

The consensus sequence of tRNA Arginine anticodons in all sequenced species is N_34_C_35_G/U_36_ [21]. Seven modified nucleotides have been identified at position 34 to date in addition to the canonical A, C, G and U. G, U and pseudo U have been observed at position 36, and finally, position 35 displays exclusively C nucleotides. Unsurprisingly, the distribution of arginine identity elements closely follows the pattern of conserved nucleotides in the anticodon. In *S*. *cerevisiae*, substitutions UA34 and UC34 are silent in tR3 transcripts. Conversely, mutations of C35 and G/U36 drastically affect the aminoacylation rate of tR3 transcripts [14].

Based on these observations, we focused our effort on C35 and G36 in tR4 and excluded position 34 due to its high variability. In addition, the crystal structure of yArgRS complexed to tR2 confirms that the interaction network stabilizing nucleotide 34 is not engaged in any base-specific contacts and that the same arrangement could virtually accommodate any nucleotides [18]. We substituted C35 and G36 individually in tR4 for the three other nucleotides and generated six different anticodons by site directed mutagenesis: CAG, CGG, CUG, CCA, CCC and CCU (mutated nucleotides are underlined).

**Fig 4:**
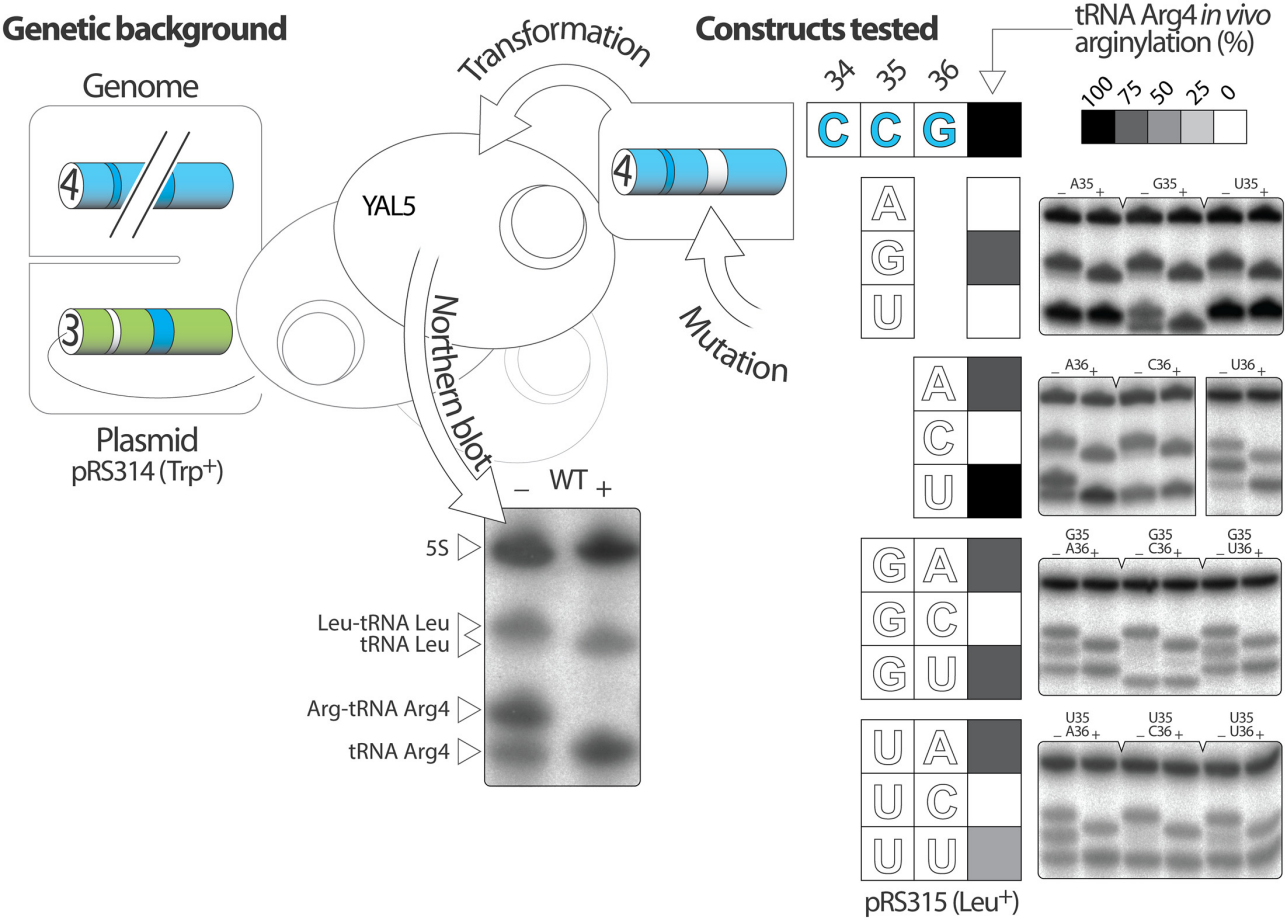
Systematic assessment of nucleotide 35 and 36: quantification of *in vivo* aminoacylation levels by Northern blot. The right panel displays the twelve tR4 anticodon mutants that were prepared, expressed in YAL5 and analyzed by Northern blot. Total RNAs were extracted and separated under acidic conditions before being transferred onto a nylon membrane and probed with a mixture of ^32^P-labeled oligonucleotides complementary to control (5S rRNA and tRNA Leu) and target (tR4 mutants) RNAs. Acylated (+) and deacylated (-) samples were loaded on separate lanes. Radioactive signals were quantified by phosphorimaging in order to estimate the cellular aminoacylation level of tR4 mutants relative to wild-type tR4. Relative *in vivo* arginylation levels are reported as shades of grey on the heat map. Arginine and leucine transferred on their cognate tRNAs, affect the migration to different extents. At pH 4.5, arginine has a net positive charge that significantly slows down the migration of tRNA Arg, yielding a wider shift. The left panel shows a modified YAL5 obtained after substituting pAL5 by a pRS314 plasmid expressing the chimeric tR3 competent for the translation of CGG codons but unable to hybridize a tR4-specific probe. This strain, free of endogenous tR4, enables the specific detection of tR4 mutants by Northern blot. Note: the actual substitution of pAL5 performed after the transformation with the mutants and before the analysis by Northern blot, is not depicted on this figure.

YAL5 screens tRNA mutants based on their ability to decode CGG arginine codons. By definition, this tool prohibits manipulating the anticodon, as any substitutions in this area would lead automatically to a red phenotype regardless of the variant’s cellular aminoacylation status. YAL5 is however a versatile tool that is not restricted to screening purpose. Building on YAL5’s favorable genetic background, we previously developed a strategy to measure specifically the arginylation level of tR4 mutants by Northern blot [15]. The same sequential approach was used in the current study to analyze the impact of anticodon mutations (Fig 4). First, YAL5 was independently transformed with the plasmids (PRS315) encoding the anticodon mutants. As anticipated, all six variants presented a solid red phenotype. Second, pAL5 encoding wild-type tR4 was substituted for a plasmid-encoded chimeric tR3 able to translate CGG codons (pRS314), but unable to hybridize a tR4-specific probe. This step cleared YAL5 transformants of endogenous tR4, enabling specific detection of our tR4 mutants by Northern blot. Within 72 hours, cells that were initially red, turned white indicating successful substitution. Finally, cells from white sectors, expressing tR4 mutants and cleared of wild-type tR4, were cultured to A_700_ = 2 in order to obtain the appropriate biomass for RNA extraction and Northern blot analysis.

Total RNAs were phenol-extracted and separated on denaturing polyacrylamide gel at 4°C under acidic conditions, which limit the hydrolysis of the arginine—tRNA ester bond [22]. RNAs were then transferred on a membrane and tR4 mutants were hybridized with a ^32^P-end labeled DNA probe specific to their 3’ end (20-mer) (Fig 4). In addition, the RNAs on the membrane were co-hybridized with two radioactive control probes specific to 5S rRNA and tRNA Leu [15, 23]. Target and control signals were quantified by phosphorimaging. Absolute aminoacylation levels of wild type and mutant tR4 were estimated as the ratio of the acylated tRNAs (upper band) by the total signal (upper + lower band). To conveniently evaluate the impact of each mutation, Northern blot results were expressed as relative aminoacylation level compared to native tR4 (mutant/WT x 100) and represented by shades of grey. On the heat map, black corresponds to optimal arginylation whereas white corresponds to a complete lack of aminoacylation. The apparent full aminoacylation level of tRNA Leu across the different samples confirmed the quality of the acidic extraction and separation. Similarly, comparable 5S signals confirmed equivalent loading. Absolute arginylation level of endogenous tR4 was estimated at 64%.

None of the mutants showed any aberrant expression pattern such as overexpression or down regulation as anticipated for mutations outside the A and B boxes. Mutants CA35, CU35 and GC36 are not substrate of yArgRS *in vivo*. Conversely, mutants GA36 and GU36 are aminoacylated at their optimal levels and CG35 only slightly affects arginylation. Interestingly, CG35 mutant presented a singular migration pattern compared to the five other variants suggesting a difference in conformation or an anomaly in post-transcriptional modifications. The migration of our acidic gels was performed at 4°C, a suboptimal temperature potentially preventing full tRNA denaturation despite the presence of 8M urea. Alternatively, the altered anticodon sequence may have scrambled the recognition signals for a modification enzyme, perturbing the pattern of post-transcriptional modifications and possibly impacting the overall tRNA molecular weight and/or charge [15].

Overall, position 36 is more flexible than 35 in terms of mutations confirming the initial prediction based on nucleotide conservation. G36 accommodates two nucleotide substitutions whereas C35 accepts only one *in vivo*. This finding supports the previous observations that the catalytic activities of tR3 transcripts displaying equivalent substitutions at position 35 are one order of magnitude lower than those at position 36 [14]. However, the near optimal *in vivo* arginylation of tR4 harboring CGG or CCA anticodons conflicts with the established model of anticodon recognition by yArgRS.

### yArgRS harnesses the malleability of the anticodon loop

Previous reports demonstrated that a suppressor tRNA Arg displaying a CUA anticodon is aminoacylated *in vivo* and *in vitro* by *E*. *Coli* ArgRS despite the absence of the major arginine identity element C35 [2, 24]. It was originally stated that the binding site on *E*. *coli* ArgRS, dedicated to the recognition of C35 and G/U36, could somehow recognize C34 and U35. Such mechanism had to rely on a partial remodeling of the anticodon loop, enabling C34 and U35 to slide in place of nucleotide 35 and 36 respectively. Implicitly, this mechanism required the cooperative sliding of the nucleotide originally at position 36.

To extend this model to yArgRS and probe the relevance of nucleotide 36, we designed 6 double mutants to generate a complete set of tR4 mutants presenting CGN or CUN anticodons (Fig 4). Three anticodons out of eight completely block the arginylation of the corresponding tR4 *in vivo*: CGC, CUC and CUG (tested previously). Four anticodons have only a moderate impact on arginylation: CGA, CGG (tested previously), CGU and CUA. Finally, the aminoacylation level of the mutant displaying a CUU anticodon is reduced by two fold compared to wild type tR4. Intriguingly, CGC and CUG anticodons induce similar migration anomalies (see interpretation above).

Our data demonstrates that the sliding of identity elements observed in *E*. *coli* also occurs in yeast. Like its bacterial homolog, yArgRS recognizes CG and CU dinucleotides as arginine identity elements regardless of their position in the anticodon (34–35 or 35–36). The malleability of this system however reaches its limit when a C stands at position 36. In tR4, C36 apparently locks the anticodon in a non-recognizable conformation for yArgRS. In addition, from the systematic analysis of CGN and CUN anticodons, it appears that G35 is more efficient at triggering the slide of identity elements than U35.

The spatial ambivalence of these determinants relies on the highly flexible nature of the anticodon loop, a feature highlighted in previous studies. The crystal structure of yArgRS complexed to tR2 shows major distortions of the anticodon loop favoring the exposure of arginine determinants to the C-terminal domain of the enzyme [18]. Upon complex formation, tR2 spreads the anticodon nucleotides towards the protein by stacking A37 between C32 and the base pair G31-C39. Increased Mg^2+^ concentrations were also shown to exacerbate the intrinsic flexibility of the anticodon *in vitro* and *in vivo* [15]. Under such conditions, it was proposed that the tRNA anticodon loop could stretch towards yArgRS in order to restore the enzyme-tRNA interaction network that was severed by mutations affecting the C-terminal domain. To a greater extent, both yArgRS and tRNA Arg have been shown to undergo dynamic conformational changes upon binding [25–27].

Finally, it should be noted that the current data set on the different tRNA Arg isoacceptors shows some significant inconstancies. For example, the catalytic activity of tR3 transcripts displaying CG, CA, CU35 and GC36 are reduced by 4920, 3330, 2670 and 123 fold respectively [14]. Cellular and *in vitro* data overlap for mutations CA35 and CU35 but conflict for CG35 and GC36, which support near full-arginylation in tR4 *in vivo*. Similarly, UC20 described above, is detrimental exclusively in tR2 but silent in tR1, 3 and 4. These observations strongly suggest that yArgRS relies on different mechanisms to recognize tR2, 3 and 4.

### Exclusive arginylation of tR4 anticodon mutants displaying ambiguous identity elements

By delocalizing the arginine identity elements in the anticodon of tR4, we fortuitously engineered two scaffolds displaying strong signals for non-cognate aminoacyl-tRNA synthetases. Yeast threonyl-tRNA synthetase (yThrRS) relies on the recognition of G35, U36 and the base pair G1-C72 to aminoacylate threonine tRNAs [28]. Likewise, tryptophanyl-tRNA synthetase (yTrpRS) recognizes C34 and C35 [29]. ThrRS and TrpRS could theoretically compete with yArgRS for the aminoacylation of the tRNA mutants presenting CGU and CCA anticodons respectively.

Observing minute misacylation on acidic gels is challenging especially in the background of strong arginylation levels. To test whether these mutants were substrates for yThrRS and yTrpRS, the corresponding transcripts were prepared and tested for the charging of ^14^C-labeled threonine and tryptophan in presence of a yeast total proteins extract. No misaminoacylation was detected under these conditions whereas the same transcripts incorporated ^14^C-arginine at rates comparable to wild type tR4 (WT tR4: 1 s^-1^, CGU: 0.25 s^-1^ and CCA: 0.86 s^-1^). Trp and Thr determinants transplanted in tR4 are not sufficient to support tryptophanylation and threonylation respectively, suggesting the presence of conflicting signals on the tR4 scaffold for the recognition by TrpRS and ThrRS. Interestingly, aspartate determinants grafted on the same scaffold support aspartylation *in vitro* [30].

### Aberrant chimeric tRNAs, mistranslation and cellular fitness

Another fortuitous consequence of the delocalization of the arginine identity elements in tR4, is the generation of five scaffolds that are arginylated *in vivo* and which anticodons are complementary to codons specific to different amino acids. tR4 mutants harboring CCA, CGG, CGA, CGU and CUU are Trp, Pro, Ser, Thr and Lys compatible decoders respectively. Such hybrid constructs are theoretically picked up by elongation factors and delivered in the A site of the ribosome as any endogenous tRNAs [31]. In addition, the ever-growing body of work involving suppressor and orthogonal tRNAs indicates that species combining tRNA scaffold and anticodon of different specificities are not actively discriminated by the A site of the ribosome [32, 33].

**Fig 5:**
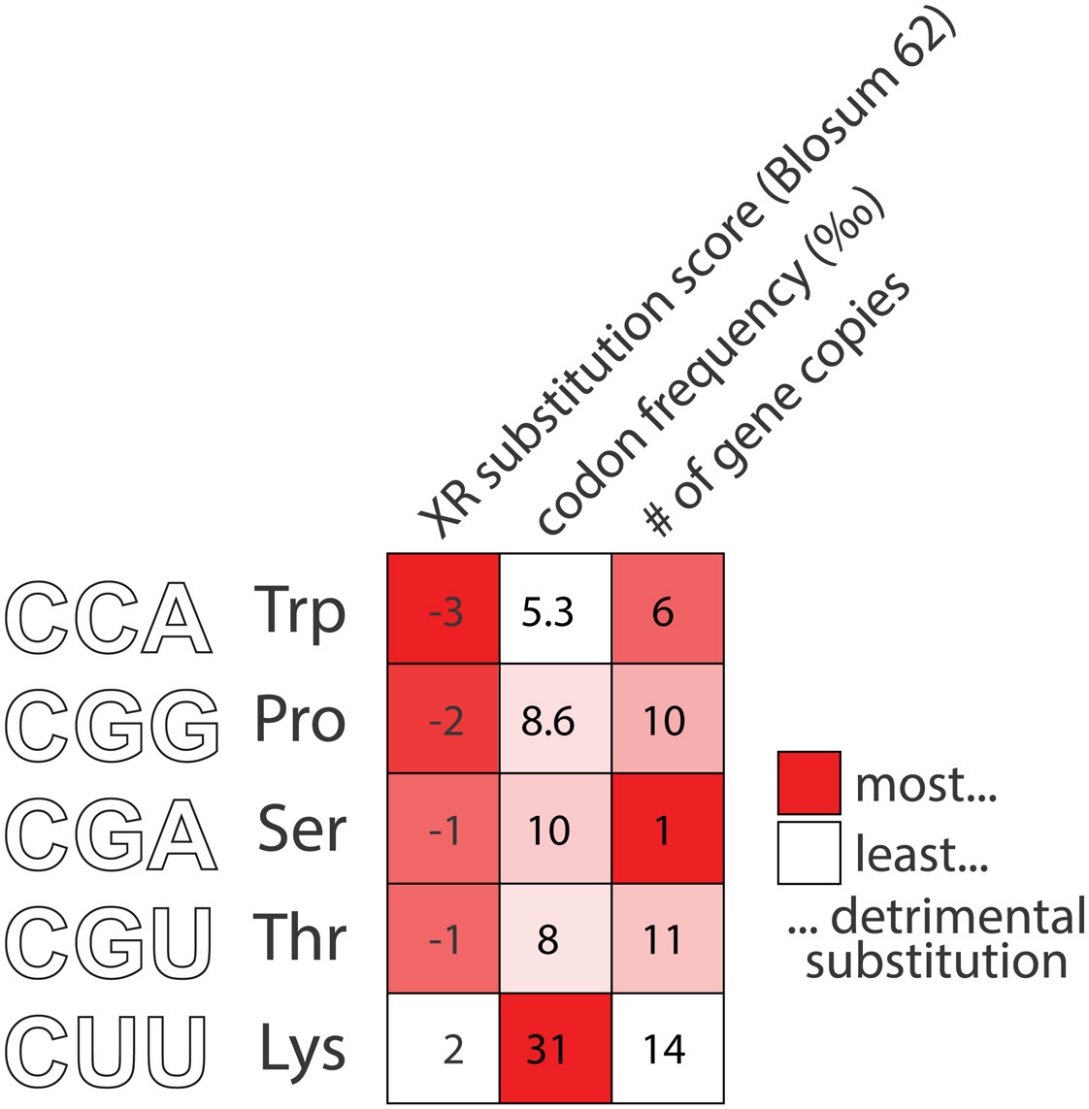
Potential impact of the cellular expression of mistranslator tRNAs. Three parameters influence arginine misincorporation in the proteome in response to the expression of mistranslating chimeras: codon representation in the genome, nature of the substitution, and the relative amount of mutant versus endogenous tRNA competing for the same codon. Gene copy number and BLOSUM 62 scores were used as a proxy for tRNA expression and substitution assessment respectively. Shades of red indicate potential toxicity on the heat map. A chimera competing with a low abundant endogenous tRNA for the mistranslation of a highly represented codon into an unfavorable amino acid, represents one of the most toxic scenarios.

As demonstrated by Northern blot, a tRNA gene, whether naturally encoded by nuclear genomic DNA or artificially cloned in a centromeric plasmid such as pRS314 or pRS315, supports tRNA expression at similar level. Therefore, tRNA variants expressed at physiological levels could compete with endogenous tRNAs for the decoding of cellular mRNAs and introduce statistical mutations in the proteome. The impact of aberrant chimeras on the quality of the proteome depends mainly on three parameters: codon representation in the genome, nature of the substitution and finally relative amount of mutant versus endogenous tRNA competing for the same codon [34]. Interestingly, none of the five mistranslator tRNAs showed a dominant lethal phenotype despite significant toxic attributes (Fig 5). The corresponding YAL5 transformants also grew at an predicted uniform rate on solid media.

## Conclusion

This work scrutinized the relevance of the tRNA nucleotides at position 20, 35 and 36 in the yeast arginylation reaction. Position 20 was systematically assessed across the four tRNA arginine isoacceptors. Arginine determinants in the anticodon were exclusively evaluated in the tR4 scaffold. The data presented here were collected *in vivo* in the previously engineered YAL5 strain. Altogether, our observations refine the pre-existing model for tRNA recognition by yArgRS that was established *in vitro* and *in silico*. Our method integrates fundamental cellular parameters such as the presence of post-transcriptional modifications as well as the notion of competition for the access to aminoacyl-tRNA synthetases. Also tRNA variants expressed inside the cell are processed and constrained by molecular competition like any other endogenous tRNAs. Our cellular approach also uniquely ties the arginylation reaction to the translation pathway and, to some extent, the entire cellular fitness. Silencing arginine determinants in the anticodon implied generating tRNA Arg species with potentially new charging and decoding specificities. The impact of such manipulations could only be addressed through cellular assays.
